# Institutional Defalcations: The Assistant Secretary's Responsibility

**Published:** 1914-06-20

**Authors:** 


*To the Editor of* The Hospital.

### Institutional Defalcations: The Assistant Secretary's Responsibility.

*To the Editor of* The Hospital.

Sir,—The letter in which "Committeeman" seeks enlightenment on the subject of the responsibility of an assistant hospital secretary for defalcations by his chief raises a question of very great moment to which I do not doubt that you will receive a variety of replies. There will almost certainly be amongst those who read "Committeeman's" letter last week many whose acquaintance with the inner workings of particular hospitals and of voluntary institutions in general is more intimate than any that I can pretend to. But at the risk of being corrected by the experts I would advance the suggestion that a hard-and-fast ruling is inadvisable and of no value.

There may be, and, as a matter of fact, I do not doubt that there are, institutions in which an assistant secretary with his wits about him and a proper degree of pride in his work could scarcely remain in ignorance of dishonesty on the part of his chief. Such an officer, if he failed to take steps to protect the institution, would be guilty either of criminal connivance with fraud or of gross carelessness such as should rightly bar him from promotion. But surely there are many institutions where this does not hold good and in which no chance is afforded him of checking the actions of his superior officer?

To suggest that in such an event the junior's chances of promotion should be jeopardised for ever and ever seems to me impolitic as well as unjust. The test thus suggested, namely, that based on the circumstances of the particular case, may be difficult to apply in certain institutions; for borderline cases are inevitable. But this judging of cases on their merits, and not by any immutable rule, is that which alone appeals as fair and reasonable to yours faithfully, Senex.

